# Kearns Sayre Syndrome (KSS) - A Rare Cause For Cardiac Pacing

**Published:** 2011-02-07

**Authors:** P Gobu, B Karthikeyan, Arun Prasath, S Santhosh, J Balachander

**Affiliations:** Department of Cardiology, Jawaharlal Institute of Post Graduate Medical Education and Research, Puducherry- 605006

**Keywords:** Kearns Sayre Syndrome, complete heart block

## Introduction

Kearns Sayre syndrome is a mitochondrial myopathy with onset before the age of 20 years [1]. It is a rare syndrome characterized by the triad of progressive external ophthalmoplegia, pigmentary retinopathy and cardiac conduction system disturbances [2]. We report a patient with KSS in whom permanent pacemaker was implanted.

## Case Report

 A 22 year old male presented to our clinic with history of fatigue and breathlessness for four years. His clinical history had started during early childhood and was characterized by mental retardation. Mental retardation had prevented him from attending the school. Patient developed bilateral ptosis when he was thirteen years old. He was also giving history of double vision for the past 6 years. Unilateral  hearing loss set in for the past 3 years. Patient started developing the symptoms of dysphagia and regurgitation for the past 2 years. The troubling symptoms which brought him to the hospital now were tiredness and easy fatigability with breathlessness.

**Figure 1:**
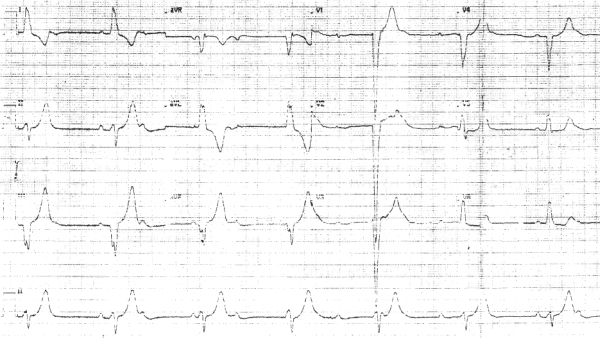
Complete heart block with a ventricular escape rhythm of 40 beats per minute

His electrocardiogram (ECG) revealed complete heart block with a ventricular escape rhythm of 40 beats per minute (Figure 1). His chest radiograph was unremarkable. Ophthalmic examination was suggestive of pigmentary retinopathy in both the eyes along with ptosis and restriction of movement of extra ocular muscles. Audiogram showed a sensorineural hearing loss of the left ear. Patient was detected to have diabetes mellitus and he required insulin for tight control of blood sugar. His thyroid status was normal. Serum electrolytes like calcium, phosphorous and magnesium were within normal range. Histopathological examination of the right deltoid muscle revealed ragged red fibers and a diagnosis of mitochondrial myopathy with clinical features of Kearns Sayre syndrome was made. Echocardiogram revealed normal chambers, normal valves, and normal LV and RV systolic function.

**Figure 2:**
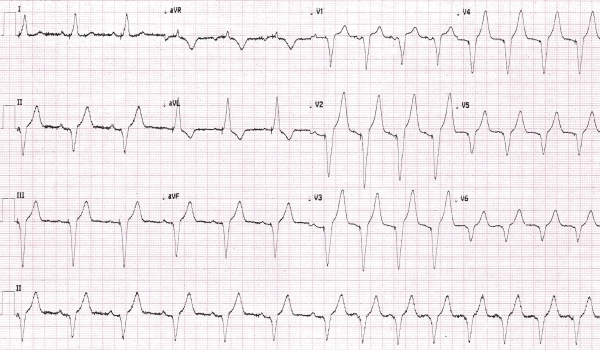
Regular pacing at a rate of 60/minute

As patient was having complete heart block, we decided to go ahead with permanent pacemaker implantation. A Medtronic Adapta DDD pacemaker was implanted through left subclavian vein puncture. Post pacing patient had a regular atrial and ventricular pacing at a rate of 60/minute (Figure 2). Patient was discharged on insulin therapy and now is on follow up with Cardiology, Neurology and Endocrinology departments. His effort tolerance and generalized malaise has significantly improved following pacemaker implantation 

## Discussion

Kearns Sayre syndrome is a rare condition characterized by the triad of external ophthalmoplegia, pigmentary retinopathy and progressive degeneration of cardiac conduction system [1].Cardiac manifestations occur in 57% of patients with Kearns Sayre syndrome which also include syncopal attacks, heart failure and cardiac arrest [3]. The most important prognostic factor in KSS patients is the involvement of heart [1], characterized by progressive degeneration of the conduction system. The various ECG abnormalities reported include third degree AV block, complete and incomplete right bundle branch blocks, fascicular blocks, and nonspecific intra ventricular conduction delays [4]. Ventricular tachyarrhythmia in the presence of normal or prolonged QT interval has also been observed[5]. Patients with KSS who have ventricular conduction defects show a rapid progression to complete heart block with an associated mortality rate of 20% [6]. Electrophysiological studies have shown an increase in His to ventricular activation time with further increase during atrial pacing [6]. The threshold for permanent pacemaker implantation in Kearns Sayre syndrome is very low because of the rapid progression of involvement of conduction tissue. Because of the progressive nature of the disease, prophylactic pacing is indicated even in patients with fascicular blocks[7]. Our patient presented with most of the features of Kearns Sayre syndrome and there was no doubt on the indication for pacing in our patient. There was a significant improvement in the general condition of the patient following pacing. His exercise capacity has improved from his baseline level. 

Involvement of the endocrine system is also common in Kearns Sayre syndrome. Diabetes mellitus, hypothyroidism and hypoparathyroidism have been reported in the literature [8]. Though our patient had diabetes, his thyroid and parathyroid status were normal but this has to be followed up continuously

Screening of family members is also essential considering the genetic nature of the disease [1]. Ventricular dysfunction has also been reported as a part of the neuromuscular diseases though our patient had a normal LV function. But our patient has to be followed progressively for any involvement of cardiac muscle [9]. Also there is a high chance of myocardial dysfunction because of the continuous pacing from the right ventricular apex. Hence our patient must be progressively monitored for LV function regularly 

In conclusion, Kearns Sayre syndrome is a rare multi system disorder with predominant involvement of the cardiac conduction system. Early prophylactic pacing is essential because of the progressive nature of the disease and pacing improves survival and symptoms of the patient. Regular and long term cardiovascular follow up is essential.
